# NLOS Identification and Positioning Algorithm Based on Localization Residual in Wireless Sensor Networks

**DOI:** 10.3390/s18092991

**Published:** 2018-09-07

**Authors:** Jingyu Hua, Yejia Yin, Weidang Lu, Yu Zhang, Feng Li

**Affiliations:** 1 Department of Electronic Engineering, Zhejiang Gongshang University, Hangzhou 310018, China; 2 Department of Communication Engineering, Zhejiang University of Technology, Hangzhou 310023, China; 13758223512@163.com (Y.Y.); luweid@zjut.edu.cn (W.L.); yzhang@zjut.edu.cn (Y.Z.); fenglzj@zjut.edu.cn (F.L.)

**Keywords:** wireless localization, non-line-of-sight error, localization residual, wireless sensor network

## Abstract

The problem of target localization in WSN (wireless sensor network) has received much attention in recent years. However, the performance of traditional localization algorithms will drastically degrade in the non-line of sight (NLOS) environment. Moreover, variable methods have been presented to address this issue, such as the optimization-based method and the NLOS modeling method. The former produces a higher complexity and the latter is sensitive to the propagating environment. Therefore, this paper puts forward a simple NLOS identification and localization algorithm based on the residual analysis, where at least two line-of-sight (LOS) propagating anchor nodes (AN) are required. First, all ANs are grouped into several subgroups, and each subgroup can get intermediate position estimates of target node through traditional localization algorithms. Then, the AN with an NLOS propagation, namely NLOS-AN, can be identified by the threshold based hypothesis test, where the test variable, i.e., the localization residual, is computed according to the intermediate position estimations. Finally, the position of target node can be estimated by only using ANs under line of sight (LOS) propagations. Simulation results show that the proposed algorithm can successfully identify the NLOS-AN, by which the following localization produces high accuracy so long as there are no less than two LOS-ANs.

## 1. Introduction

Target localization in the wireless sensor network has received immense attention in recent years. According to the measured localization parameter, the target localization can be classified as: time of arrival (TOA) [1,2,3], time difference of arrival (TDOA) [4,5], received signal strength indication (RSSI) [6,7,8], angle of arrival (AOA) [9], as well as their mixing parameters TOA/AOA [10], TDOA/AOA [11] and RSS/AOA [12,13]. But regardless of localization parameters, the localization accuracy will be affected by two major errors. The one is the measurement error, which is usually defined as a Gaussian variable of zero mean [14]. The second is the non-line of sight (NLOS) error caused by the refraction and reflection during signal propagation. In practice, the latter is much larger than the former, and has become a crucial factor for the positioning [14,15]. In a wide area network, such as the macro cellular network, NLOS error may approaches several hundreds meters [16], while in the small area network, such as the WSN, it may covers tens of meters. Therefore, the NLOS error suppression has become a key issue in the wireless localization.

In order to tackle the NLOS issue, there are three kinds of suppression algorithms. The first is trying to model the non-line-of-sight error, and then employ this model to finely estimate the position of target note (TN) [17], such as RSS-based positioning method [18,19,20,21]. These methods are usually sensitive to the propagating environment. However, it is difficult to obtain a precise as well as universal NLOS model for complex real-world environments, thus such algorithms are difficult to be widely used. The second algorithms attempt to weight the measured distance or the intermediate position estimates to get a better positioning performance, where the weight plays an important role. These weights may be relate to the geometric relationship as well as algebraic relationship between ANs and TN, and then a typical choice is to construct the weighting function with residual parameters [22]. However, such algorithms can not eliminate the impact of large NLOS errors, and then some robust algorithms that treat NLOS bias as nuisance parameters, are derived according to the optimization theory [1,2,3,23]. Unfortunately, the optimization method improves the positioning performance at the cost of significantly increased complexity. The final class of algorithm identifies the NLOS-AN and then uses only LOS-AN for position estimation [8,12,13]. The advantage of such an algorithm is the high accuracy in case of successful identification of all LOS-ANs, while the determination of detection threshold is an essential and difficult issue. In summary, a simple as well as effective NLOS suppression algorithm still requires further study, which is the target of our study.

According to the above discussions, this paper proposes an algorithm belonging to the third category, where two LOS-ANs are analogous to [13]. In general, the proposed algorithm is based on a least-squares criterion, which makes it very light in terms of computational complexity. The detail processing of proposed algorithm can be summarized as follows. First, we group all ANs into two-AN subgroups. Then, we obtain three intermediate position estimates for each subgroup, and calculate the residual according to these intermediate estimates. Here the residual is defined as the distance between two position estimates. Second, a hypothesis test is proposed according to the localization residual, in which the threshold is determined by an analytical plus simulated way. Such a threshold determination is simple and effective compared with previous schemes. Accordingly, in line with the comparison of threshold and residual, the NLOS-AN can be identified. Finally, we can estimate the TN position using only LOS-ANs. In fact, if we do not detect two LOS-ANs, we will use all ANs to do the least-squares localization. We verify our study by computer simulations, and the results demonstrate the high identification accuracy of NLOS-AN. Then the final TN position estimation also produces high accuracy. Note that only two LOS-ANs are necessary for the proposed algorithm.

The rest of this paper is organized as follows. The second section presents the system model and the third section derives the proposed algorithm. The simulation and analysis are introduced in the fourth section and conclusions can be found in the fifth section.

## 2. System Model

In our study, we assume that the sensor node has the ability to estimate TOA and AOA, but the specific estimation methods are not our goal. Moreover, analogous to conventional localization studies, we also assume that both TOA and AOA estimates have been obtained, and these estimates are corrupted by the measurement noise and NLOS error. Besides, we can employ the range measurement and angle measurement of both ANs to localize the TN by some positioning methods. If a node wants to use RSS to calculate the distance, our method is not applicable.

**Figure 1:**
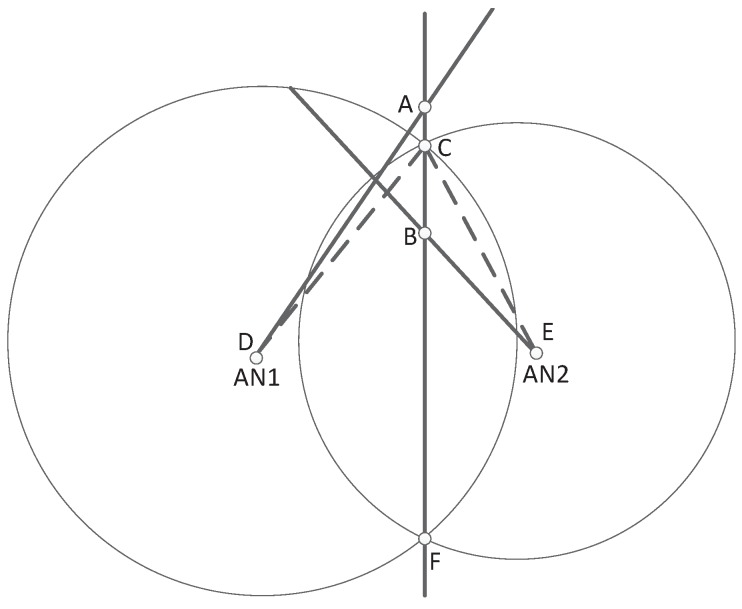
The diagram for localization geometry.

A simplified diagram can be found in Figure 1, where the TN and its two adjacent *AN*s, i.e., *AN*_1_ and *AN*_2_, are presented. In Figure 1, the line $\bar{AF}$ denotes the intersection line of two circles centered at $AN_{1}$ and $AN_{2}$, where the circle radiuses equal to measured distances between ANs and TN. Then the measured AOA line of $AN_{1}$ ($\bar{AD}$) intersects $\bar{AF}$ at the position A, which can be treated as an intermediate position estimate of TN. Similarly, the position B represents the intermediate position estimate for the measured AOA line of $AN_{2}$. In addition, the circle intersecting point C can be treated as another intermediate position estimation of TN. Obviously, due to the measurement noise and NLOS errors, positions {A, B, C} do not coincide.

Assuming that the coordinates of target and anchors are (x,y) and $\left(x_{i},y_{i}\right)$, we can write the distance between TN and ANs as [24].

(1) $$r_{i}^{2}=K_{i}-2x_{i}x-2y_{i}y+x^{2}+y^{2}$$

where $K_{i}={x_{i}}^{2}+{y_{i}}^{2}$. Moreover, the AOA of $AN_{i}$ can be shown as

(2) $$\tan\theta_{i}=\frac{y-y_{i}}{x-x_{i}}$$

Without loss of generality, we denote ANs of a subgroup as $AN_{1}$ and $AN_{2}$, and then have the following distance equations:

(3) $$\left\{\begin{array}{c} r_{1}^{2}=K_{1}-2x_{1}x-2y_{1}y+x^{2}+y^{2}\\ r_{2}^{2}=K_{2}-2x_{2}x-2y_{2}y+x^{2}+y^{2} \end{array}\right.$$

From above equations, one can obtain the line $\bar{AF}$ in Figure 1:

(4) $$r_{1}^{2}-r_{2}^{2}=K_{1}-K_{2}+2\left(x_{2}-x_{1}\right)x+2\left(y_{2}-y_{1}\right)y$$

Moreover, the formula to find the coordinates of point A is shown as follows:

(5) $$\left\{\begin{array}{c} r_{1}^{2}-r_{2}^{2}-K_{1}+K_{2}=2\left(x_{2}-x_{1}\right)x+2\left(y_{2}-y_{1}\right)y\\ y_{1}\cos\theta_{1}-x_{1}\sin\theta_{1}=y\cos\theta_{1}-x\sin\theta_{1} \end{array}\right.$$

The formula to find the coordinates of point B is shown as follows:

(6) $$\left\{\begin{array}{c} r_{1}^{2}-r_{2}^{2}-K_{1}+K_{2}=2\left(x_{2}-x_{1}\right)x+2\left(y_{2}-y_{1}\right)y\\ y_{2}\cos\theta_{2}-x_{2}\sin\theta_{2}=y\cos\theta_{2}-x\sin\theta_{2} \end{array}\right.$$

The above two equations can be transformed into the matrix form, such as for point A:

(7) $$Y=AX_{A}$$

with $Y=\left[\begin{array}{c} r_{1}^{2}-r_{2}^{2}-K_{1}+K_{2}\\ y_{1}\cos\theta_{1}-x_{1}\sin\theta_{1} \end{array}\right]$, $A=\left[\begin{array}{cc} 2(x_{2}-x_{1}) & 2(y_{2}-y_{1})\\ -\sin\theta_{1} & \cos\theta_{1} \end{array}\right]$, $X_{A}=\left[\begin{array}{c} x\\ y \end{array}\right]$. Then the least-squares (LS) estimation of position A can be shown as

(8) $${\hat{X}}_{A}={\left(A^{T}A\right)}^{-1}A^{T}Y$$

Similarly, the coordinates of possition B can also be obtained by solving a least-squares problem with respect to (6). Then, the position residual is defined as the distance between point A and point B.

(9) $$\Delta AB=\bar{AB}$$

Previous studies indicated that different localization methods produced adjacent position estimates in the LOS environment [25], i.e., the distance between position estimates were comparable to the standard deviation of ranging noise. By contrast, these position estimates must be obviously deviated from each other in the NLOS environment, resulting in much larger position residuals in comparison with those of LOS scenarios. Moreover, as pointed out above, point A, point B and point C can be treated as TN position estimates produced by different localization methods. Therefore, in the LOS environment, the distance between A and B, the distance between A and C, and the distance between B and C will all be relatively small, i.e., several times of standard deviation of ranging noise. Then, we can define two more residuals as

(10) $$\begin{array}{c} \Delta AC^{\prime }=\min\left(\bar{AC},\bar{AF}\right)\\ \Delta BC^{\prime }=\min\left(\bar{BC},\bar{BF}\right) \end{array}$$

where the symbol $C{}^{\prime }$ represents the point of {C, F} closer to point A, i.e., $C^{\prime }=\left\{\begin{array}{c} C,\;\mathrm{if}\;\bar{AC}⩽\bar{AF}\\ F,\;\mathrm{if}\;\bar{AC}>\bar{AF} \end{array}\right.$.

## 3. The Proposed NLOS-AN Identification and Localization

### 3.1. System Error Analysis

Before the detail derivations, we must point out that our derivation is in the sense of statistic, but the expectation symbol is omitted for simplicity. Furthermore, we assume that measurement noises of range and angle have small variances. Now, if there is only measurement noise, we have the perturbed version of (7)

(11) $$Y^{0}+\Delta Y=\left(A^{0}+\Delta A\right)\left(X_{A}^{0}+\Delta X_{A}\right)$$

where ${\left(•\right)}^{0}$ and Δ(•) denote the actual value of variable and the corresponding perturbed parts.

Equation (11) can be further expanded as

(12) $$Y^{0}+\Delta Y=\left(A^{0}+\Delta A\right)\left(X_{A}^{0}+\Delta X_{A}\right)=A^{0}X_{A}^{0}+\Delta {\mathrm{AX}}_{A}^{0}+A^{0}\Delta X_{A}+\Delta A\Delta X_{A}$$

Since $Y^{0}=A^{0}X_{A}^{0}$, Formula (12) can be simplified as

(13) $$\Delta Y=\Delta {\mathrm{AX}}_{A}^{0}+\left(A^{0}+\Delta A\right)\Delta X_{A}$$

Thus, the estimation error caused by measurement error can be derived as

(14) $$\Delta X_{A}={\left(A^{0}+\Delta A\right)}^{-1}\left(\Delta Y-\Delta {\mathrm{AX}}_{A}^{0}\right)$$

Since we have assume small measurement noises in the LOS environment, we have approximations as $\Delta r_{1}^{2}\approx \Delta r_{2}^{2}$, $\sin\Delta \theta_{1}\approx \Delta \theta_{1}$ and $\cos\Delta \theta_{1}\approx 1$. Then, we can derive $\Delta Y\approx \left[\begin{array}{c} 2r_{1}\Delta r_{1}-2r_{2}\Delta r_{2}\\ -\Delta \theta_{1}y_{1}\sin\theta_{1}-\Delta \theta_{1}x_{1}\cos\theta_{1} \end{array}\right]$, $\Delta A\approx \left[\begin{array}{cc} 0 & 0\\ -\cos\theta_{1}\Delta \theta_{1} & -\sin\theta_{1}\Delta \theta_{1} \end{array}\right]$. Note that the expressions of ΔY and ΔA can also be explained by the differential theory.

Similarly, it is also possible to find the error $\Delta X_{B}$ of point B, then the deviation between point A and point B can be computed as

(15) $$\Delta d=∥\begin{array}{c} \Delta X_{A}-\Delta X_{B}\; \end{array}∥$$

where ∥•∥ denote the norm operation.

By applying triangular inequality, we have that $∥\begin{array}{c} \Delta X_{A}-\Delta X_{B}\; \end{array}∥\leq ∥\begin{array}{c} \Delta X_{A}\; \end{array}∥+∥\begin{array}{c} \Delta X_{B}\; \end{array}∥$, then the threshold can be defined as:

(16) $$\Lambda =∥\Delta X_{A}∥+∥\Delta X_{B}∥$$

From (15) to (16), it is suggested that if the estimating error is less than a threshold, the included two ANs are highly possible to be LOS-ANs. In fact, our next simulation confirms this prediction. Otherwise, there is at least one NLOS-AN. However, if we use (16) as the threshold, $X_{A}^{0}$ is unknown. Though $X_{A}^{0}$ can be replaced with the TN position estimation (${\hat{X}}_{A}$) of (8), the AOA measurement is highly instable in the NLOS environment, which significantly affects the matrixes $\left\{{\left(A^{0}+\Delta A\right)}^{-1},\Delta Y,\Delta A\right\}$. Finally, Formula (16) may yield a large and loose threshold, resulting in an increased missing probability of NLOS-AN detection. Hence, we will show in the next section how to resolve this issue.

### 3.2. Threshold Determination and NLOS-AN Identification

In order to tackle the above problem, we look for principles from the geometric relation, and then propose a novel threshold. From Figure 1, it is clearly that the maximal distance between point A and point B occurs when points A and B are on the upper and lower sides of point C, and then we have

(17) $$\Delta AB=\bar{AB}=\bar{AC}+\bar{CB}$$

When there is no NLOS propagation, the AOA measurement error is trivial, i.e., ∠ADC and ∠BEC are small. Denoting these two angles as $\omega_{1}$ and $\omega_{2}$, we have the following trigonometric expression

(18a) $$\begin{array}{c} \bar{AC}\approx r_{1}^{0}*\omega_{1} \end{array}$$

(18b) $$\begin{array}{c} \bar{BC}\approx r_{2}^{0}*\omega_{2} \end{array}$$

Assuming the same standard deviation of AOA measurement, we can derive the following expression

(19a) $$\begin{array}{c} \Delta AB\leq r_{1}^{0}*\omega_{1}+r_{2}^{0}*\omega_{2}\leq \Lambda_{1} \end{array}$$

(19b) $$\begin{array}{c} \;\Lambda_{1}=\lambda \sigma_{a}\left(r_{1}^{0}+r_{2}^{0}\right) \end{array}$$

where $\sigma_{a}$ denotes the standard deviation of AOA measurement. If the parameter λ is large enough, the inequation will be satisfied with a high possibility. Unfortunately, this single residual is not enough for the NLOS-AN identification. For example, a large λ increases the missing probability of NLOS-AN detection. Hence, we introduce other two residuals as detection variables

(20a) $$\begin{array}{c} \Delta AC=\bar{AC}\approx r_{1}^{0}*\sin\omega_{1}\approx r_{1}^{0}*\omega_{1}\leq \Lambda_{2} \end{array}$$

(20b) $$\begin{array}{c} \;\Lambda_{2}=\lambda \sigma_{a}*r_{1}^{0} \end{array}$$

(20c) $$\begin{array}{c} \Delta BC=\bar{BC}\approx r_{2}^{0}*\sin\omega_{2}\approx r_{2}^{0}*\omega_{2}\leq \Lambda_{3} \end{array}$$

(20d) $$\begin{array}{c} \;\Lambda_{3}=\lambda \sigma_{a}*r_{2}^{0} \end{array}$$

where $\sin\omega_{k}\approx \omega_{k}$ holds for small $\omega_{k}$ in the LOS environment. We must indicate that the derivations of (17)∼(20) are not sensitive to relative positions of {A, B, C}, i.e., the proposed algorithm still works even if the point A is below the point B.

In real-world applications, the actual values used in (19)∼(20) are unknown, and we have to replace them by measured values. Since λ can be determined artificially, the influence of this substitution will be compensated by searching appropriate λ. Then, the final residual detector can be derived as:

(21) $$\left\{\begin{array}{c} \Delta AB\leq \Lambda_{1}\;\mathrm{and}\;\Delta AC\leq \Lambda_{2}\;\mathrm{and}\;\Delta BC\leq \Lambda_{3}\Rightarrow \mathrm{Both}\;\mathrm{LOS-ANs}\\ \Delta AB>\Lambda_{1}\;\mathrm{or}\;\Delta AC>\Lambda_{2}\;\mathrm{or}\;\Delta BC>\Lambda_{3}\Rightarrow \mathrm{at}\;\mathrm{least}\;\mathrm{one}\;\mathrm{NLOS-AN} \end{array}\right.$$

However, there are three comparisons in (21), leading to large thresholds to satisfy three inequations at the same time. Simulations demonstrate that two comparisons are enough. Therefore, the detector (21) can be reformulated as

(22) $$\left\{\begin{array}{c} \mathrm{Satisfy}\;\mathrm{at}\;\mathrm{least}\;\mathrm{two}\;\mathrm{of}\;\{\Delta AB\leq \Lambda_{1},\Delta AC\leq \Lambda_{2},\Delta BC\leq \Lambda_{3}\;\;\;\}\;\;\;\Rightarrow \mathrm{Both}\;\mathrm{LOS-ANs}\\ \mathrm{Satisfy}\;\mathrm{at}\;\mathrm{most}\;\mathrm{one}\;\mathrm{of}\;\{\Delta AB\leq \Lambda_{1},\Delta AC\leq \Lambda_{2},\Delta BC\leq \Lambda_{3}\;\;\;\}\;\;\;\Rightarrow \mathrm{at}\;\mathrm{least}\;\mathrm{one}\;\mathrm{NLOS-AN} \end{array}\right.$$

A complete NLOS-AN identification and localization process can be summarized as:

(1) Find the coordinates of points A and B using (8), and then solve the circle intersections C and F.
(2) Calculate the lengths of AC and AF separately, then find the nearest point $C^{{}^{\prime }}$ of point A from {C,F}.
(3) Treat {A, B, C’} as three intermediate position estimates.
(4) Use $C^{{}^{\prime }}$ to calculate ΔAC as well as ΔBC, and finally compute ΔAB.
(5) Identify the NLOS-AN by the detector (22).
(6) Estimate the TN position by using LOS-ANs only.

In the above step (6), after combining range and angle measurements of LOS-ANs, we can construct a mixed positioning equation as:

(23) $$A_{L}X=Y_{L}$$

(24) $$A_{L}=\left[\begin{array}{ccc} -2x_{1} & -2y_{1} & 1\\ & \vdots & \\ -2x_{M} & -2y_{M}, & 1\\ -\tan\theta_{1} & 1 & 0\\ & \vdots & \\ -\tan\theta_{M} & 1 & 0 \end{array}\right],X=\left[\begin{array}{c} x\\ y\\ R \end{array}\right],Y_{L}=\left[\begin{array}{c} r_{1}^{2}-x_{1}^{2}-y_{1}^{2}\\ \vdots \\ r_{M}^{2}-x_{M}^{2}-y_{M}^{2}\\ y_{1}-\tan\theta_{1}x_{1}\\ \vdots \\ y_{M}-\tan\theta_{M}x_{M} \end{array}\right]$$

where $R=x^{2}+y^{2}$ and *M* represents the number of LOS-ANs. The above equation can be solved by least squares algorithm, such as

(25) $$\hat{X}={\left({A_{L}}^{T}A_{L}\right)}^{-1}{A_{L}}^{T}Y_{L}$$

Note that if we cannot identify at least two LOS-ANs, we will estimate the TN position according to (8), i.e., operate the LS localization by exploiting all ANs.

In the NLOS-AN identification mentioned above, an important mission is to determine the λ and therefore the threshold, which will be presented next.

## 4. Simulation and Analysis

### 4.1. Threshold Determination

**Figure 2:**
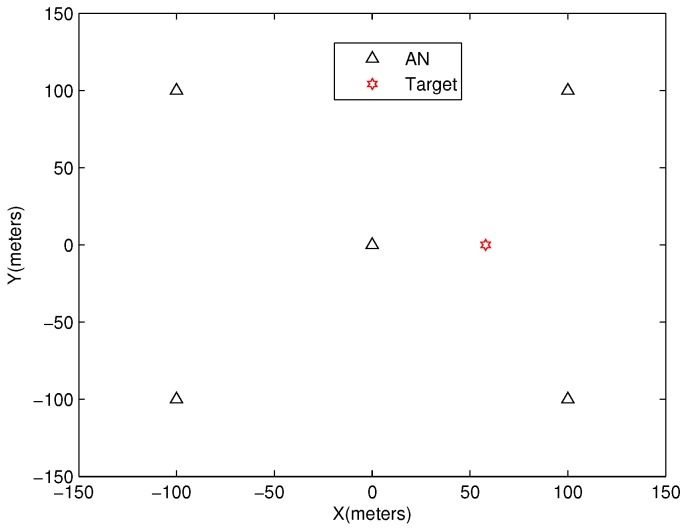
AN topology.

There are five ANs located in a 100-m × 100-m area as shown in Figure 2, and their locations are (0, 0), (100, 100), (−100, 100), (−100, −100) and (100, −100). Without loss of generality, the TN is placed at (58, 0), and the range and angle measurements usually satisfy

(26) $$\begin{array}{c} {\hat{r}}_{k}=r_{k}^{0}+e_{k}^{NLOS}+e_{k}^{noise} \end{array}$$

(27) $$\begin{array}{c} {\hat{\theta }}_{k}=\theta_{k}^{0}+z_{k}^{NLOS}+z_{k}^{noise} \end{array}$$

where k∈{1,2,3,4,5}. Moreover, both the range measurement noise ($e_{k}^{noise}$) and the angle measurement noise ($z_{k}^{noise}$) are zero-mean Gaussian variables determined by their standard deviations. Besides, the NLOS error of range ($e_{k}^{NLOS}$) is uniformly distributed between 10 m and 50 m, whereas the NLOS error of angle ($z_{k}^{NLOS}$) obeys the uniform distribution between $-180^{{}^{\circ }}$ and $180^{{}^{\circ }}$. From (26) and (27), we explicitly see the process to generate measurements. First, we should generate the TN position, and then we can compute $r_{k}^{0}$ as well as $\theta_{k}^{0}$ according to the geometric topology. Second, we generate the NLOS errors according to their statistical distributions. Subsequently, the Gaussian noises are generated according to their statistical distributions. Finally, we combine all terms according to (26) and (27).

**Figure 3:**
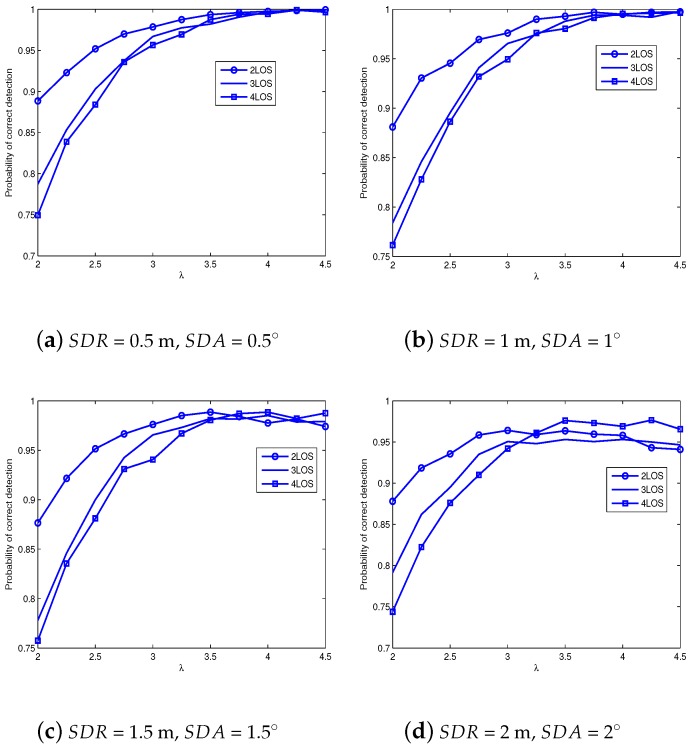
Threshold analysis.

Here we must determine the reasonable threshold based on Formulae (18)–(20). Since Formulae (18)–(20) are only semi-analytical derivations, the simulations are required to final determine λ and therefore the thresholds, which will be shown in Figure 3. Figure 3 presents the simulations of searching λ, where four typical measurement noise settings are taken into consideration, i.e., SDR (standard deviation of range) and SDA (standard deviation of angle) take value from {0.5 m, 0.5${}^{\circ }$}, {1 m, 1${}^{\circ }$}, {1.5 m, 1.5${}^{\circ }$} and {2 m, 2${}^{\circ }$}. Note that the last setting represents serious measurement noises, which may improve the robustness of proposed threshold.

From Figure 3, when {SDR = 0.5 m, SDA = 0.5${}^{\circ }$} or {SDR = 1 m, SDA = 1${}^{\circ }$}, the NLOS-ANs can be easily identified with a high threshold. However, as the measurement error increases, a high threshold is prone to identification failure. Therefore, we can combine all feasible regions of four subfigures, and then determine the final threshold according to the intersection of feasible regions. Accordingly, if the target of detection probability is 0.95, Figure 3a–d respectively produce the feasible regions as λ≥3.1, λ≥3.2, λ≥3 and 3.2≤λ≤4.25. Thus the final feasible region is 3.2≤λ≤4.25. Subsequently, a fine search of λ in the range [3.2, 4.25] reveals that λ=4 is an appropriate threshold, which will be further confirmed by the next positioning simulations.

### 4.2. Analysis for the Positioning Performance

After the NLOS-AN identification, we will operate the localization by the simple LS method, where only identified LOS-ANs are included so long as there are at least two identified LOS-ANs. As seen in Figure 3, the identification probability is large, which definitely produces superior positioning performance as shown in next simulations. Moreover, we also choose some classical or latest alorithms as comparisons, as described in Table 1.

**Figure 4:**
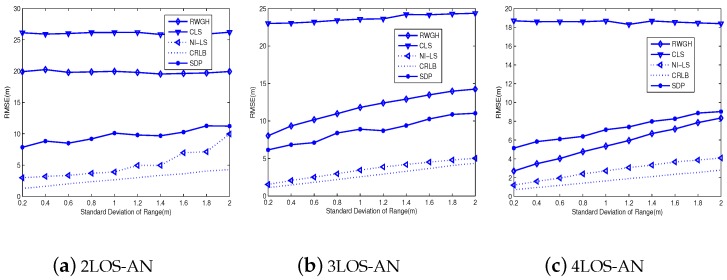
The influence of SDR on the accuracy: $SDA=1{}^{\circ }$.

**Figure 5:**
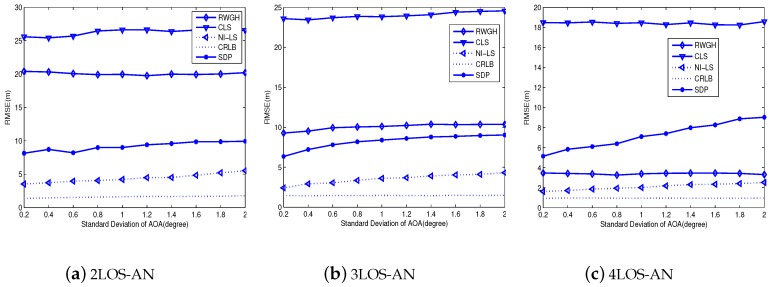
The influence of SDA on the accuracy: SDR = 0.5 m.

Figure 4 and Figure 5 compare the root-mean-square-errors (RMSE) for the tested algorithms. From Figure 4, we explicitly see the best performance of the proposed algorithm, and the gap between it and CRLB is small. Moreover, with the increase of LOS-AN, the RMSE of all tested algorithms tends to decrease. Note that the gap between the CRLB and the proposed algorithm is small and tends to be invariant, so long as the number of LOS-AN is larger than four. In fact, this gap of 3LOS-AN is only trivially larger than that of 4LOS-AN. On the other hand, the SDP method is relatively stable to the LOS-AN number, which make it lose its advantage in the case equipped with more LOS-ANs. Meanwhile, the SDP method uses the optimization tool, thus its computational complexity is higher than the non-optimization method, such as the proposed algorithm and the RWGH method. In addition, the RWGH algorithm produces great improvements with the increase of LOS-AN number, which is because it uses three-AN subgroups and benefit from the increase of LOS-AN number. From Figure 5, we can find conclusions agree with those from Figure 4. Furthermore, we have seen from Figure 5 that the angular deviation does not have a great impact on the positioning accuracy, so long as the SDA is within a reasonable range. In fact, the proposed algorithm produces a slowly rising RMSE w.r.t SDA, and approximately linear curves suggest good performance for even larger SDAs. On the other hand, we must indicate that even the current AOA configuration yields a large AOA error. First, the NLOS error of AOA is equally possible in the range of −180∼$180^{\circ }$, resulting in a large value in general. Second, the measurement noise of AOA is a Gaussian variable, which may produce a large value even for SDA = $2^{\circ }$.

**Figure 6:**
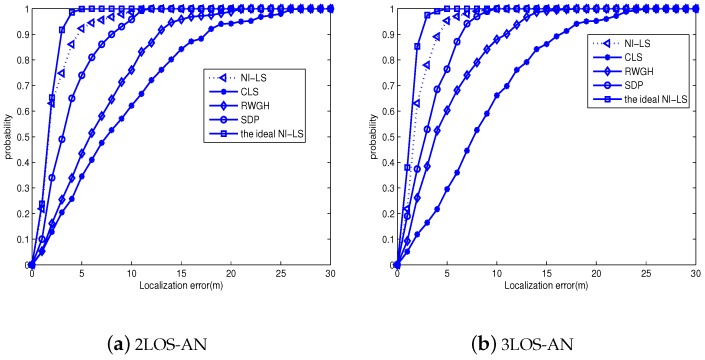
The cumulative distributed function (CDF) of tested algorithms: SDR = 2 m, $SDA=1{}^{\circ }$.

Figure 6 presents comparisons for the CDF performance at SDR = 2 m, SDA = 1${}^{\circ }$, i.e., a typical measurement noise level in existing literature [28,29]. From it, we definitely see the superior performance of the proposed algorithm, i.e., even with only two LOS-ANs, the NI-LS method yields about nine meters errors with probability higher than 0.95. When the LOS-AN number approaches three, this error reaches about six meters with probability higher than 0.95. Moreover, there is still gap between the NI-LS and ideal NI-LS, which must caused by the identification failure. The reason is that the λ is determined to cover wide ranges of SDR and SDA, hence it is not optimal for a certain setting of SDR and SDA, resulting in some identification failure. However, the excellent positioning accuracy indicates that the identification accuracy is high. Meanwhile, the decreasing CDF gap between the NI-LS and ideal NI-LS suggests that the NLOS-AN identification tends to be more and more accurate by exploiting more LOS-ANs.

**Figure 7:**
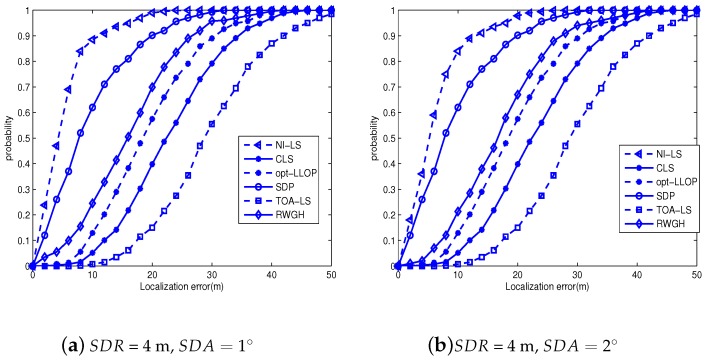
The CDF of tested algorithms under harsh environments: 2LOS-AN.

In order to test the proposed algorithm in more realistic and harsh environments, we present new simulations in Figure 7, where the NLOS error of range is uniformly distributed within 20∼70 m. Moreover, the SDR reaches four meters and only two LOS-ANs are employed. From Figure 7, we clearly see that the proposed algorithm significantly outperforms its counterparts. Moreover, we also see the performance degradation in comparison with Figure 6, which means that the harsh environment deteriorates localization performance. However, the proposed algorithm still produces acceptable CDF, i.e., it is about 0.9 when the localization error is eleven meters. Therefore, the proposed algorithm produces enough performance redundant to combat the harsh environment.

Summarizing the above discussions, we must point out that the correct NLOS-AN identification is important for wireless localization. Fortunately, the proposed algorithm produces excellent detection performance even in very harsh environments, and then it yields superior positioning performance in comparison with its localization counterparts that do not distinguish between LOS/NLOS links.

## 5. Conclusions

The Non-line-of-sight error significantly reduces the localization accuracy, therefore this paper proposes a two-step algorithm to suppress the influence of NLOS errors, where at least two LOS-ANs are required. First, we define a novel residual addressing the positioning difference. Second, a threshold hypothesis test is employed to identify the NLOS-AN. Finally, the target position is estimated by all identified LOS-ANs. We test the proposed algorithm by computer simulations, and results show a good and superior performance. In conclusion, the proposed algorithm can effectively identify the NLOS-AN and significantly improve the positioning accuracy under the hybrid NLOS/LOS environment.

## Figures and Tables

**Table 1:** Various algorithms and their description.

Algorithm	Description
RWGH	Residual weighting algorithm [25]
CLS	Constrained Least Squares Algorithm [26]
NI-LS	Using the least squares algorithm after NLOS-AN identification
Ideal-NI-LS	Using the least squares algorithm with known LOS-AN
CRLB	Cramer-Rao lower bound (CRLB) with known LOS-AN [24]
SDP	Convex semidefinite programming algorithm [4]
opt-LLOP	Linear optimization algorithm [27]
